# The impact of increasing land productivity on groundwater dynamics: a case study of an oasis located at the edge of the Gobi Desert

**DOI:** 10.1186/s13021-020-00142-7

**Published:** 2020-05-02

**Authors:** Wu Lei, Li Changbin, Xie Xuhong, He Zhibin, Wang Wanrui, Zhang Yuan, Wei Jianmei, Lv Jianan

**Affiliations:** 1 Key Laboratory of Western China’s Environmental Systems (Ministry of Education), College of Earth and Environmental Sciences, Lanzhou University, No. 222 South Tainshui Road, Chengguan District, Lanzhou, 730000 Gansu Province China; 2 Northwest Institute of Eco-Environment and Resources, Chinese Academy of Sciences, Lanzhou, 730000 China; 3 National Key Laboratory of Desert and Oasis Ecology, Xinjiang Institute of Ecology and Geography, Chinese Academy of Sciences, Urumqi, 830011 China

**Keywords:** Oasis water utilization, Regional AET, Groundwater responses, Shiyang River Basin

## Abstract

**Background:**

Intensification of agricultural systems may result in overexploitation of water resources in arid regions because enhanced productivity of crops is often associated with increased actual evapotranspiration (AET). The aim of this study was to quantify the effect of increased regional AET on the groundwater level in a case study of the oasis located within the Shiyang River Basin near the edge of the Gobi Desert.

**Result:**

The results of the study show that regional AET increased during the period from 1981 to 2010 due to increasing oasis area and air temperature. The water losses due to AET exceeded the water supply from the mountainous discharges of the basin by the end of this period, leading to groundwater overexploitation in the oasis area.

**Conclusions:**

This case study shows the importance of considering the effect of climate change on water losses associated with increasing agricultural production for the sustainable agricultural development of arid regions.

## Background

Anthropogenic modifications have had substantial influences on natural earth systems in past centuries. For example agricultural development has led to a significant impact on water-soil exploitation [1], resulting in remarkable variations in regional ecohydrology and the availability of water resources in many parts of the world [2–6]. Today, human beings are facing a series of water-related challenges, including the need to meet growing requirements for food production, human health, environmental safety, and so on [7, 8]. The inherent requirement of water resources for socioeconomic development, especially in arid or semiarid regions, has caused river exploitation to reach capacity [9], resulting in deterioration of the river systems and groundwater systems [10–12], as well as degradation of land surface ecology [13, 14]. Specifically, inland river basins in northwestern China have experienced prosperous periods over the past thousands of years when the availability of water was adequate for a limited amount of agriculture and grazing [15]. The balance of supply and demand in many natural-artificial water systems in this region has sharply tipped in recent decades due to rapid population growth and oasis scale expansion [16, 17]. The original nature of river-groundwater exchange in the middle and lower parts of the basins was changed by division/storage projects and groundwater pumping. Artificial procedures have greatly altered the natural horizontally dominated slower water movement [18] into vertically dominated faster processes in irrigated areas [19, 20]. Water availability could not match the speed of consumption. Stresses of continuously increasing utilization have inevitably resulted in general water overexploitation in critically water-scarce areas [21–23].

From the perspective of water balance in inland river basins, mountainous discharge maintains the existence of natural oases, artificial oases and groundwater systems [24, 25]. On the one hand, due to the arid climate and intensive evapotranspiration, rare precipitation cannot form locally effective recharge for the groundwater system in the plain area of the inland river basin [26]. On the other hand, desert vegetation is significantly affected by local precipitation, while oasis water consumption for vegetative productivity is mainly supported by mountainous discharge and groundwater [27]. With the continuous expansion of the oasis scale and the increasing consumption of water for industrial and life purposes, water resources from mountains have gradually become insufficient, and local areas have to exploit groundwater to meet the increasing water requirements [28]. Based the above information, surface runoff development leads to a decrease in supplies, and groundwater abstraction reduces reserves, both of which contribute to rapid groundwater system degradation [29].

Quantification of the ecological-environmental processes that involve water in arid regions is essential for understanding and planning local development. However, errors in data collection regarding water utilization may result in significant uncertainties in analyses. Fortunately, land surface processes, such as variation in greenness and AET monitored by satellite sensors, as well as their derivations, satisfactorily represent reality [30], e.g., classification of land use/cover types in a satellite image could be a key to determining hydrological variables, such as AET, for evaluation of anthropogenic modification impacts on complex hydrological processes [31–33]. In addition, fine-temporal imagery products, such as the NDVI (normalized difference vegetation index) from Advanced Very High Resolution Radiometer (AVHRR) or Moderate Resolution Imaging Spectroradiometer (MODIS) sensors have provided potential long-term land cover change interpretations [34–36], which is also an important widely used indicator for studying the carbon–water balance in the context of climate change and plays a key role in water management [37–41].

Given the high-level complexity of multifactor interactions in natural-artificial water systems [42], the hydrological responses to climate and human impacts across a whole basin are difficult to quantifiably predict. In particular, the quantification of the relationship between the groundwater system and oasis scale [43] in arid inland river basins has rarely been reported. In this study, a 30-year data package was prepared, which includes hydrometeorology, remotely sensed NDVI, GLEAM-AET and other data. The objective of the study was to analyze oasis changes during the 1981 to 2010 period, determine oasis vegetation water consumption using GLEAM-AET data combined with surveys of water utilization in life and industrial systems, calculate oasis water consumption and water deficit, and then assess groundwater abstraction and its effectiveness in the regional groundwater system. The study could help residents better understand the artificial impacts on arid hydrological processes and benefit water planning and watershed management in arid regions under the background of climate change.

## Method

### Study area

**Fig. 1:**
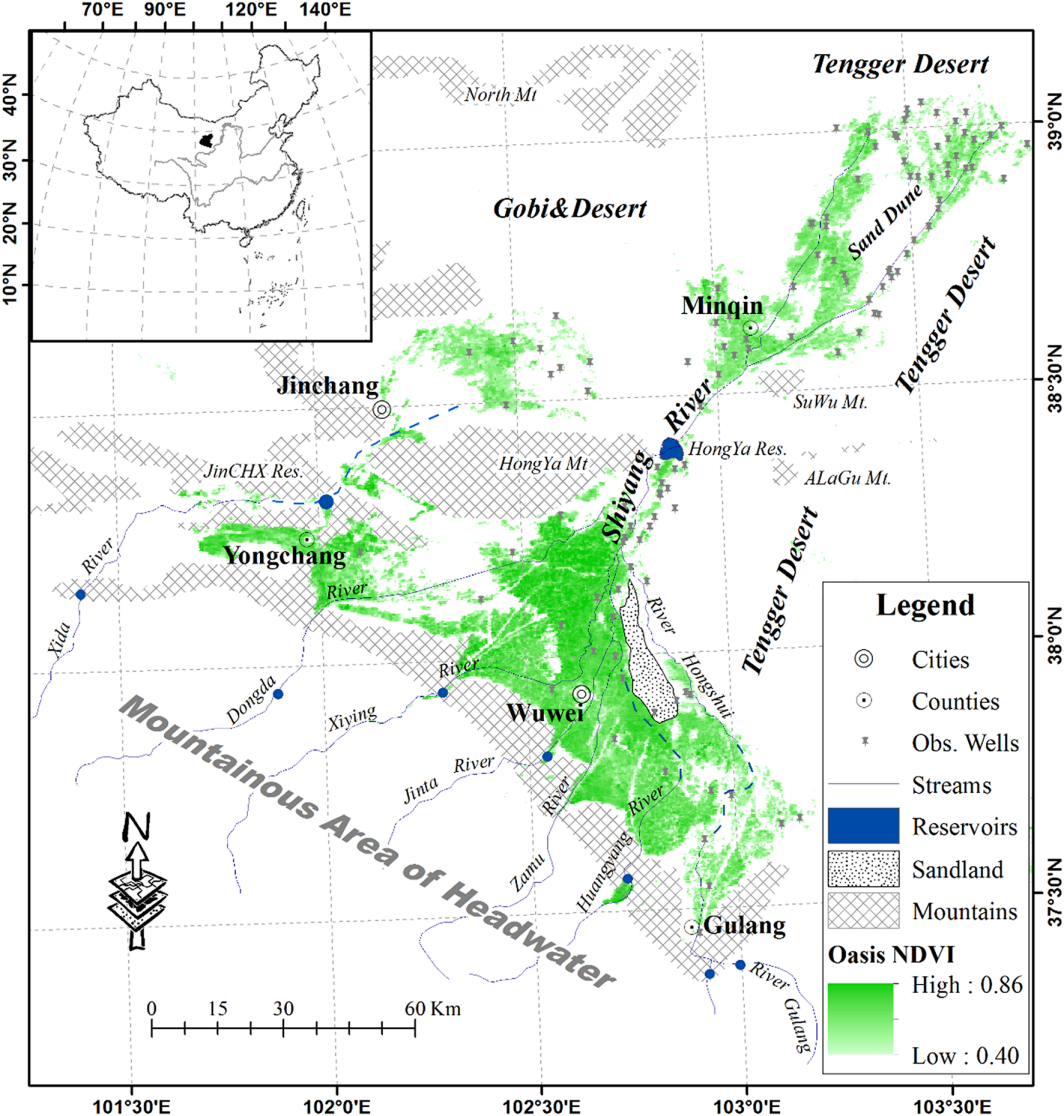
Location map and oasis distribution in the SYRB. The basin is divided into upper, middle and lower reach areas according to regional hydrogeomorphology. The oases are mainly located in the latter two reach areas, which north of the Hongya Reservoir are considered to be in the lower reaches (administratively part of the Minqin district), and others are considered to be in the middle reaches (administratively part of the Wuwei, Jingchang and Yongchang districts)

The Shiyang River Basin (SYRB) is located in the easternmost part of the Hexi Corridor, which is mainly formed by 7 upstream rivers sourced from Qilian Mountain (southwestern area shown in Fig. 1). The Shiyang River flows north and passes Hongya Mountain, flowing into the Minqin district in the lower reaches. The flow generation area is located in the alpine zones with elevations ranging from 5130 to 1855 m, and this area is dominated by lower Paleozoic epimetamorphic rocks and intensively cutting river networks. Groundwater is mainly fissure vein and interlayer water, which discharges into the river channels, flowing out of the mountainous outlets and into the middle and lower reaches where the elevation ranges between 1855 and 1260 m, from the northernmost part of the southern mountains to the edge of the northern desert. Channel river water infiltrates and percolates into the deep Quaternary alluvial fans and deep underground from 100 m to more than 200 m in the south slope area of the mountains, overflowing at the edge of the fans as springs and accumulating again in the land surface rivers; finally, this water cuts through the northern mountain valleys and partially becomes groundwater in the lacustrine deposit system. The remaining water forms ephemeral rump lakes and is eventually consumed by evapotranspiration, maintaining the regional water balance in the area [44]. Land covers show significant spatial heterogeneity, with distinct vertical zonation across the basin. The hydrological conditions in the area are representative of inland river basins [45].

At present, there are nearly 2.4 million people in the SYRB, and the population density is over 600 people per km^2^ in the oasis area. The amount of water required to match socioeconomic development in the area has dramatically increased since the early 1980s in conjunction with the extension of arable land. The irrigated area in the 1970s was approximately 0.25 million ha, which expanded to over 0.4 million ha from the 1980s to 2010s. The large demand for agricultural irrigation has increased the use of mountainous discharge and groundwater abstraction. The overexploitation of water resources has strained the regional developmental capacity for a relatively long time [46], resulting in a series of hydrological consequences, including considerable drawdown of groundwater [47]. Land surface ecology has deteriorated due to the groundwater system decline; most xerophytic shrubs, such as elaeagnus angustifolia and white spines, have died out. As desertification increased, native farmers were forced to migrate out of the area as “environmental refugees” [16].

### Data collection and processing

Hydrometeorological data were collected in or near the SYRB, including data from 10 national weather stations (China meteorological data sharing network, http://cdc.cma.gov.cn) and seven hydrological observational stations (Gansu Province Bureau of Hydrology and Water Resources Monitoring) at the outlets of the mountainous sub-basins. The double-mass curve method [48] was used to check consistency for each hydrometeorological factor. The analogy method (linear) was adopted to interpolate or extend the missing data to complete the full 30-year series from 1981 to 2010. Actual evapotranspiration (AET) data were sourced from GLEAM (Global Land Evaporation Amsterdam Model, http://www.gleam.eu) datasets with a spatial resolution of 0.25°, which have been verified to agree well with land surface flux observations in China [49–51]. Especially in arid northwestern China, which has few observation stations, GLEAM-AET can help to obtain spatially and temporally better regional AET [52, 53]. Compared with other AET products (e.g., MOD16, JRA, GLDAS), GLEAM-AET has higher spatial and temporal resolutions (0.25°, 1 day) and matches better with data requirements in basin-scale studies. The spatial distribution of different AET products was similar to some extent, although the expression of high values of MOD16 AET was too large [54], and the seasonal volatility of GLDAS AET was relatively high, especially in spring [55]. Monthly GLEAM-AET data collected over a year was accumulated into the annual GLEAM-AET from 1981 to 2010. All AET data are resampled to a 250 m resolution to maintain consistency with other data. We collected well-monitored groundwater data from administrative units, including the Gansu Province Bureau of Hydrology and Water Resources Monitoring, Gansu Province Geology Survey, and Gansu Province Environmental Protection Agency. Data from the 129 monitoring wells were mainly distributed in the oasis area. Data for the local socioeconomy and the related water utilization were sourced from official statistics. The NDVI data required for the study were derived from the AVHRR, 8 km resolution, 1981 to 2006, https://ecocast.arc.nasa.gov/) and the MODIS, 250 m resolution, 2001 to 2010, http://glovis.usgs.gov/). The land use/cover map used as the basis for calibration and validation of land cover classification was obtained from China’s National Land Cover Dataset (NLCD 2000, http://www.resdc.cn/).

### NDVI data cross-calibration

The AVHRR and MODIS NDVI products were used for determination of the maximum pixel NDVI value by the maximum-value composite (MVC) method in this study. All of the remotely sensed data were resampled into 250 m × 250 m images to keep the spatial resolution identical. Next, series consistency was assessed and cross-calibrated by correlating corresponding pixels during the overlapping time period from 2001 to 2006 [34]. A linear regression method implemented for the overlapping time period was used to assimilate the two series for the extension of data to the full time series from 1981 to 2010. The regressive equation for cross-calibration was found in the following linear form:

1 $${\text{NDVI}}_{\text{MODIS}} \; = \;0.87{\text{NDVI}}_{\text{AVHRR}} \; + \;0.21$$

The consistency of the two NDVI series was verified, with a correlation coefficient of 0.79 (P ≤ 0.05). The assimilated NDVI dataset could be considered satisfactory for series extension, and the data resolution was treated at the same level as the MODIS data.

### Variation statistics

The spatiotemporal variations in the key factors are analyzed using zonal statistics and Sen’s slope method [56]. Sen’s slope is a widely used method for variation analyses of hydrological and meteorological series. Judgment is based on the median of the series of slopes. When evaluating the variation trend and amplitude of the time series, the Sen’s slope method can reduce or avoid the impact of data anomalies and omissions. The formula is outlined as follows:

2 $${\text{Sen}}_{ij} \; = \;{\text{MEDIAN}}\frac{{\left( {X_{j} - X_{i} } \right)}}{{\left( {j - i} \right)}}$$

where Sen_*ij*_ is Sen’s slope; and X_*i*_ and X_*j*_ represent the sequential values corresponding to times i and j, respectively, where 1 < *i* < *j *< *n*, *n* is the length of the series.

### Determination of oasis extent

The area of the oasis was determined by coupling thresholds from the remotely sensed NDVI value and DTM datasets. Thresholds of vegetation greenness (NDVI value), altitude (DEM) and slope (derived from DEM) were defined according to the AOI (Area of Interest) training samples corresponding to the land cover types illustrated in an NLCD map. The above thresholds were used to determine the yearly extent of the oasis in the plain area of the SYRB.

Analysis of the AOIs resulted in thresholds of altitude ≤ 2200 m and slope ≤ 7°, corresponding to locations of arable oases that were mainly distributed in plain areas, reflecting that significant cultivation occurred in the relatively flat area with lower altitude in the SYRB. The annual maximum NDVI value based on the MVC method was trained into ≥ 0.4 in these areas, including small areas of forest and grassland with high land coverage supported by irrigation, near water bodies or in areas with shallow groundwater burial. Other land cover types, such as sparse vegetation (arid or semiarid shrubs or grassland) and bare land (desert or Gobi), were excluded because the focus of this study is mainly on artificial water utilization and its effectiveness in groundwater systems.

### Regressive module of AET

The driving mechanism of oasis AET was explored based on regressions by setting areal averages of air temperature (T), precipitation (P) and the NDVI values as independent variables under a multi-linear module at the monthly time scale. For reasonable regression fittings, AIC (Akaike information criterion) values [57] were calculated step by step under different parameter settings to assess the reliability of the model’s capacity and parameter selection. The data used to interpret the influencing mechanism of the AET dynamics is the GLEAM-AET product, which is considered to be the observed AET series. During the process of the stepwise regression, we used the whole time series (1981–2010) for exploration of the driven module:

3 $${\text{AET}}_{ } = \left( {\sum b_{i} *C_{i} } \right) + {{\upvarepsilon }}$$

where $b_{i}$ is the regression coefficient for the key climatic or vegetative index ($C_{i}$) and ${\upvarepsilon}$ is a constant term.

### Groundwater dynamics

**Fig. 2:**
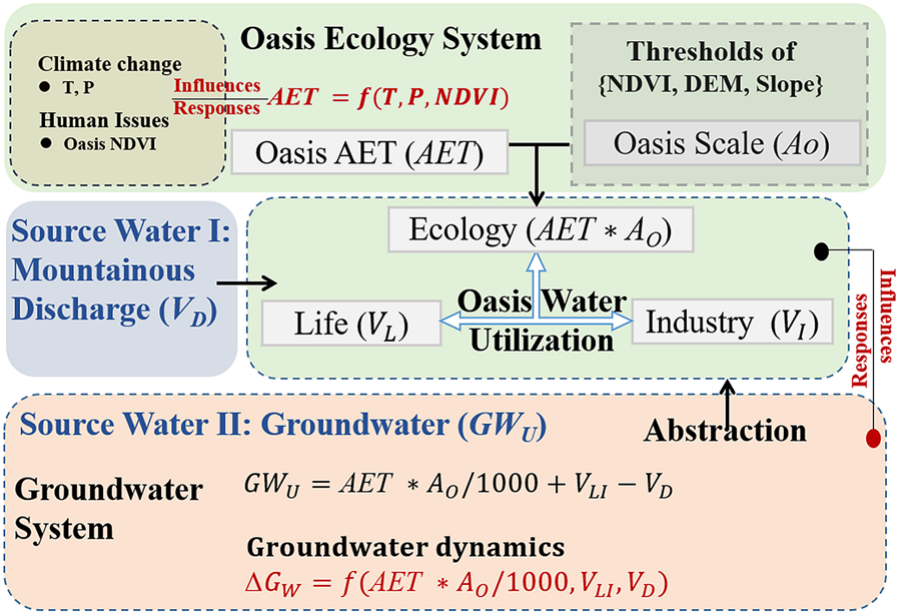
The coupling framework determining the groundwater dynamics in response to oasis water consumption through regional AET

Flooding irrigation in the study area dominated infiltration and percolation through the soil layer to the phreatic system. We assume that the recharged groundwater part would finally provide abstraction and contribute to AET consumption in the oasis area. Thus, groundwater utilization could be generally illustrated by a simple water balance module under a “net” framework (Eq. 4). Precipitation is excluded because it is rarely able to recharge groundwater systems in arid regions where precipitation is within 100–200 mm/a [47, 58, 59]:

4 $${\text{GW}}_{\text{U}} = {\text{AET}}_{ } * {\text{A}}_{\text{O}} /1000 + {\text{V}}_{\text{LI}} - {\text{V}}_{\text{D}}$$

where ${\text{GW}}_{\text{U}}$ is the annually positive/negative balance of the groundwater (10^8^ m^3^);${\text{A}}_{\text{O}}$ is the area of the oasis (10^4^ ha); ${\text{V}}_{\text{LI}}$ is the surveyed water consumption for life and industrial purposes (10^8^ m^3^); and ${\text{V}}_{\text{D}}$ is the division of mountainous discharge (Fig. 2) that supports oasis water utilization (10^8^ m^3^, which could be determined by surveying water division works at the mountain outlets and the Hongya Reservoir, which provides water to the lower area of the basin). Number 1000 is the arithmetic operator.

We consider the groundwater dynamics to be a holistic consequence influenced by land surface and underground water supply and utilization (Fig. 2). Thus, considering the observations of mountainous discharges, surveyed life and industrial water utilization and the simulated/monitored AET and ${\text{A}}_{\text{O}}$, the relationships between groundwater level dynamics and other factors could be further explored using the following regressive formation:

5 $$\Delta {\text{G}}_{\text{W}} = f\left( {{\text{AET}}_{ } * {\text{A}}_{\text{O}} /1000, {\text{V}}_{\text{LI}} , {\text{V}}_{\text{D}} } \right)$$

where $\Delta {\text{G}}_{\text{W}}$ is the accumulative dynamics of the groundwater level according to the regional average of well monitoring.

## Results

### Oasis scale from 1981 to 2010

**Fig. 3:**
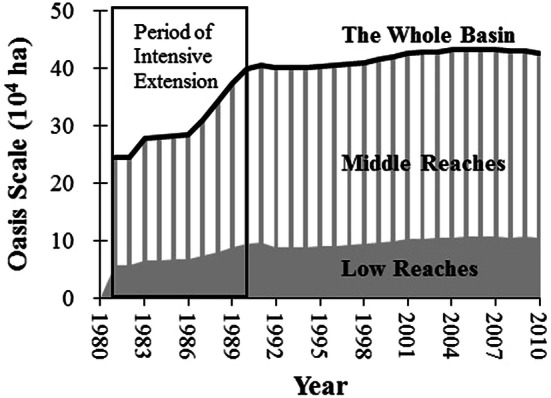
Changes in the oasis scale in the SYRB from 1981 to 2010

The scale of the oasis continued to increase during the period. Surveys and our analysis both pointed to a significant extension of the irrigated oasis scale during the 1980s (Fig. 3). The oasis area increased from 24.5 × 10^4^ ha to nearly 40 × 10^4^ ha over the ten-year period. Subsequently, the increase in oasis area became slighter than previously. In the late 2000s, a small shrinkage was found in the middle reaches, which occupied approximately 77% of the total oasis area of the basin.

### Correlation between AET and climatic and vegetative factors

#### Changes in the key variables

**Fig. 4:**
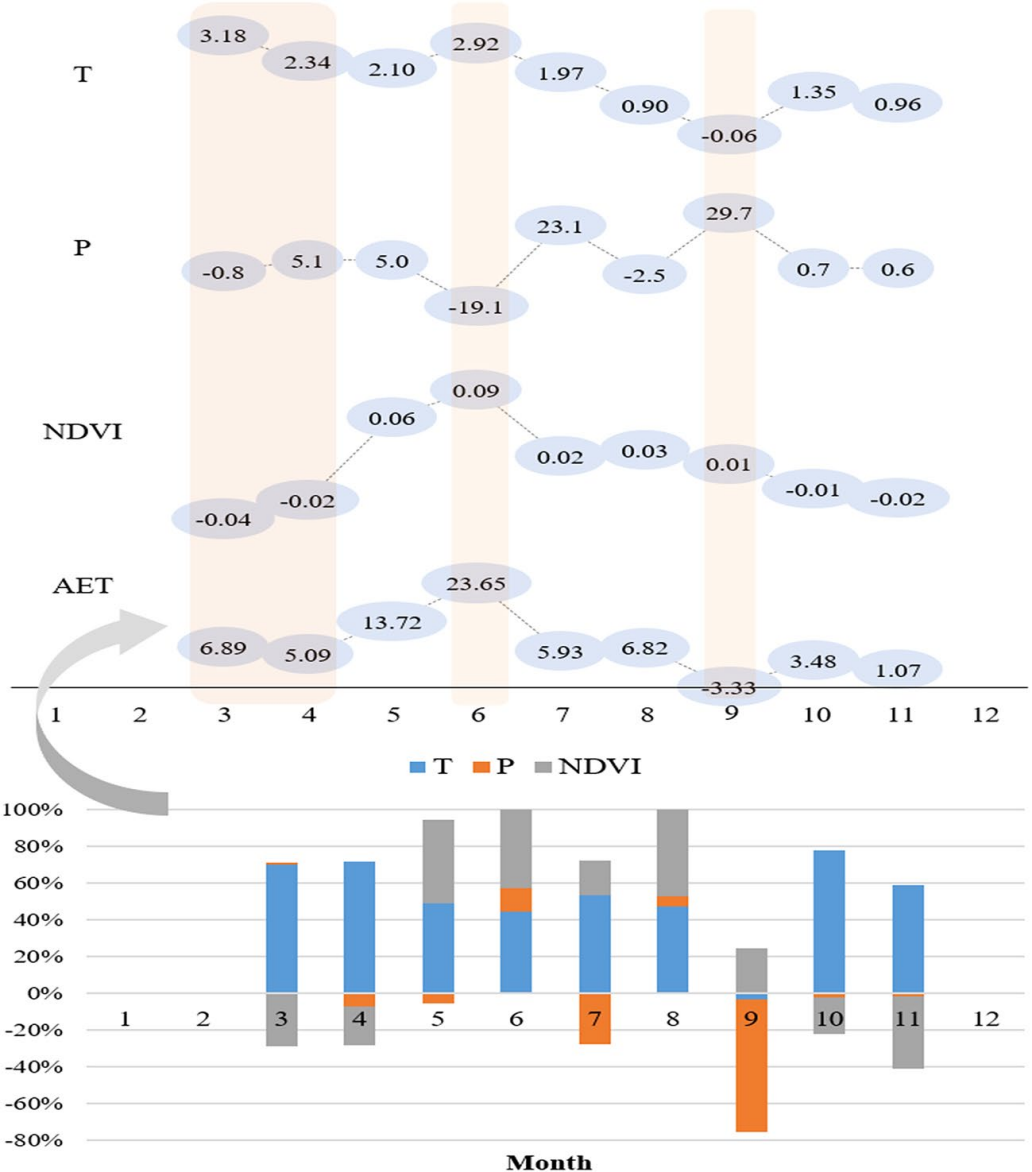
Monthly variations in each factor and their contributions to AET variation. The positive and negative signs represent the enhanced or weakened effects

Statistics of the monthly variation in the 30-year span resulted in different changeable amplitudes for all 4 variables (Fig. 4). Generally, warming facilitated aridity in arid areas because of sparse precipitation, although artificial irrigation changed the water supply and supported vegetation growth. Greenness reflected by NDVI presented suppression in most of the initial growing stages, regardless of whether the precipitation increased or decreased. The AET increased along with the upward air temperature in each stage (Fig. 4, columns 3–4), reflecting the facilitation of warming on the regional AET. Most crops in the study area turned into developing/middle stages from May to July. Statistics showed in an obvious decrease in precipitation but a remarkable increase in AET in June (Fig. 4, column 6). Given the small amount of rainfall, irrigation played an important role in this stage. Additionally, less precipitation may correspond to more sunny days, verifying the increasing AET requirement at this stage. Similarly, increased rainfall (more cloudy days), combined with the decrease in air temperature in September, suppressed AET to some extent, although the greenness (NDVI) strengthened (Fig. 4, column 9). This indicates that except for vegetative transpiration, environmental loss through soil evaporation was necessary to support the total AET water consumption.

#### Regressive module of AET

For a better understanding of AET variation under the background of climate change and human activities in the oasis area, the growing season GLEAM-AET (mainly during the time period from March to November in the area) was regressed in a stepwise manner using the key factors of T, P and NDVI, as illustrated in Eq. (3). Zonal statistics were used to calculate values of the four factors at a monthly time scale. We used the forward stepwise regression method to input factors and compared the AIC values. The AICs decreased to 994.93, 970.48, and 954.79 as the number of parameters increased from 1 to 3, respectively, indicating that the effectiveness of the selected model and variables in AET simulation is reliable. Regressions showed positive influences of T and NDVI but a negative influence of P on regional AET variations. The final module by stepwise regression was calibrated as follows:

6 $${\text{AET}}\;{ = }\; 3. 5 9\; \times \;{\text{T - 0}} . 1 6\; \times \;{\text{P}}\; + \;117.23\; \times \;{\text{NDVI - 24}} . 9 5$$

where AET represents the simulated AET in the oasis area when key climatic factors and the vegetative index are considered to be auxiliary variables, T is the average monthly air temperature (°C), P is the monthly total precipitation (mm), and NDVI is the MVC-based monthly NDVI.

**Fig. 5:**
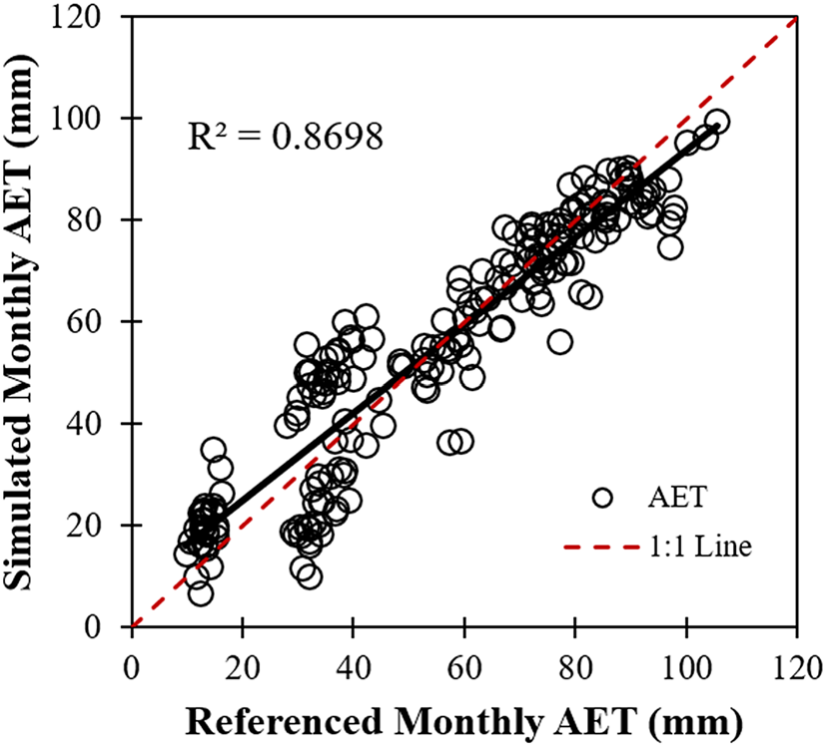
Module validation of the climate and vegetation factors driving regional AET

The stepwise regression was fitted with satisfactory precision, and the correlation coefficient was 0.87 when compared with the GLEAM-AET. (Figure 5), meaning that the regressive module could explain approximately 87% of the GLEAM-AET.

#### Relationship between AET and the influential factors

Module applications and statistics revealed that during the 1981 to 2010 period, the air temperature increased by 1.74 °C, facilitating an increase in AET by 56.2 mm (approximately 32.3 mm/ °C). The increase in precipitation during the growing season over the 30-year period was summed to 41.7 mm, with an approximately 6.7 mm suppression of AET (an approximate ratio of − 1.6 mm/10 mm). The oasis scale presented extension according to remote sensing monitoring, and the NDVI value averaged into a total increase of 0.12, resulting in an AET of an additional 13.7 mm (an approximate ratio of 11.4 mm corresponding to the strengthened unit greenness of 0.1). The above aggregated findings pointed to unit influences of 32.3 mm/ °C, − 1.6 mm/10 mm and 11.4 mm/0.1 in the changeable T, P and NDVI, respectively, on oasis AET variations in the SYRB (Fig. 9b). Consequently, the contribution ratios of climate change (varied T and P) and oasis ecology (mainly supported by irrigation) to the changed AET were calculated to be 78% and 22%, respectively, at the 30-year time scale. Although artificial irrigation basically supported the oasis ecology, it was warming that dominated the increase in the consumptive oasis AET in arid regions, as in the SYRB, given a growing vegetation condition of adequate water supply.

### Net water consumption in the oasis area of the SYRB

Net oasis water consumption was determined by the product of GLEAM-AET and the remote sensing-based delineation of the oasis scale. Spatial statistics resulted in average AET consumptions of 359.97 mm/a and 442.46 mm/a, corresponding to total oasis water utilizations of 11.04 × 10^8^ m^3^ and 3.89 × 10^8^ m^3^ in the middle and lower reaches, respectively. Combined with household and industry water consumption, averaged as 1.96 × 10^8^ m^3^ and 0.17 × 10^8^ m^3^ in the middle and lower oases during the reference period, respectively, the net water consumption in the oasis area of the SYRB was 17.06 × 10^8^ m^3^, although multi-yearly monitored mountainous discharges were averaged as a total volume of 13.99 × 10^8^ m^3^ over the 30-year period. When considering possible water loss through evaporation in rivers, wetlands and reservoirs, the total water demand far exceeded the available water resources in the SYRB.

### Responses of the groundwater system to oasis water utilization

#### Net consumption of groundwater

Due to the large water requirement in the oasis area, water utilization has continuously increased in China’s inland river basins in recent decades. At the basin level, the gross supply of water resources consists of two parts: the water amounts extracted from mountainous discharges and groundwater abstractions. According to our statistics, the reservoir-canal systems extracted almost all mountainous discharges for oasis water utilization in the middle and lower reaches, which provided approximately 82% of the total oasis water consumption; the remainder was mainly supported by local groundwater abstraction.

**Fig. 6:**
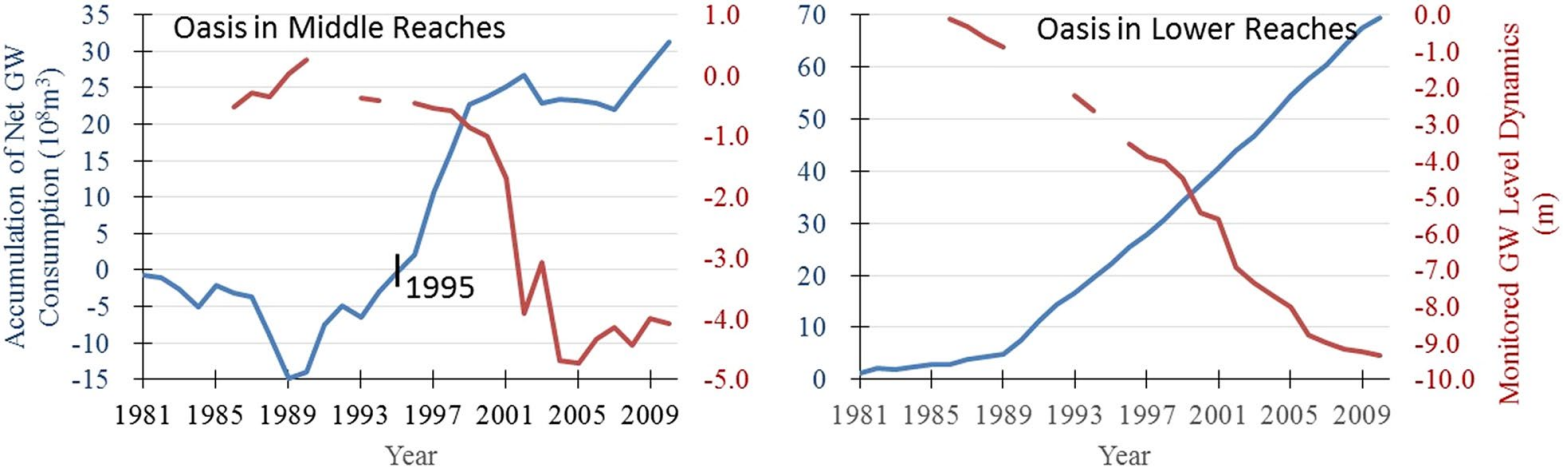
Accumulation trends of net groundwater consumption and groundwater level dynamics. Inverse trends were found for both of these factors in oasis areas in the middle and lower reaches of the SYRB

A series of simulated oasis AET and remote sensing monitored oasis scale data, combined with monitored or surveyed mountainous discharges and life and industrial water use, were input as parameters into Eq. (4) to determine the net balance of the groundwater system (${\text{GW}}_{\text{U}}$). Groundwater levels from monitoring wells (Fig. 1) were treated as differential series to illustrate the dynamics, which were spatially averaged to represent the regional responses corresponding to net groundwater consumption. Collaborative trends of the two accumulative series in both the middle and lower reaches (Fig. 6) indicated that quantification under a net balance module was reliable for estimating the influences of oasis water utilization on the groundwater system. Obvious differences in groundwater utilization and responses were found in the middle and lower reaches. More net consumption of groundwater led to near-linear and greater drawdown of the groundwater level in the lower reaches, while that in the middle oasis area fluctuated occasionally as a result of the large percolation recharge from channel transfer and irrigation. Consequently, before 1995, there was an overall positive groundwater balance in the middle part of the basin, and the total water consumption in the area was less than the mountainous discharge provided. Conversely, there was continual drawdown in the lower oasis area over the whole period, indicating a continuously negative balance in the groundwater system there. Considering water sources, the relatively smaller drawdown of the groundwater level in the area of the middle reaches benefitted from the advantages of the mountainous discharge, while the obviously severe decline in the underground water system in the lower reaches was mainly because of the finite water division into the Hongya Reservoir and the intensive abstraction.

#### Relationship between groundwater dynamics and influential factors

**Fig. 7:**
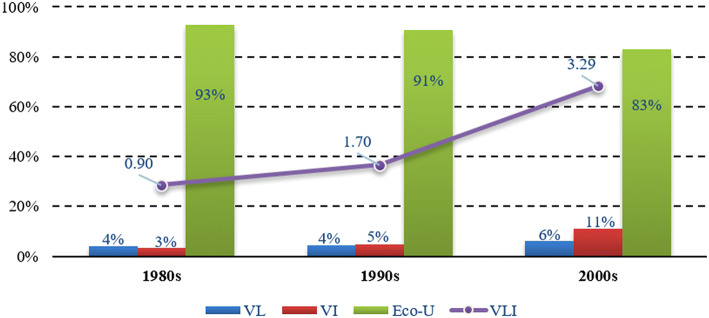
Percentages of water used for different purposes in the SYRB. (Legend: V_L_, V_I_ and Eco-U represent water use by life, the manufacturing industry and oasis consumption, respectively. V_LI_ shows the variation in life and industrial water use during different decades (in 10^8^ m^3^).)

Accumulative series of the variables in Eq. (5) were stepwise regressed to calibrate the key factors affecting the groundwater dynamics. Life and industrial water (V_LI_) was considered to be one of the potential factors influencing groundwater dynamics in the equation. This value increased from 0.9 × 10^8^ to 3.29 × 10^8^ from 1981 to 2010, although the proportions were still small (only 7–17% to the annual total, Fig. 7). V_LI_ was excluded during the regressive exploration for the relatively small fitting contributions, meaning that the groundwater dynamics were not synergetic or significant to the increase in life and industrial water use, and key factors influencing regional groundwater dynamics were mountainous discharge and oasis ecological water utilization.

Stepwise regression resulted in the following expression:

7 $$\Delta {\text{G}}_{\text{W}} = 0.138{\text{AET}}_{ } * {\text{A}}_{\text{O}} /1000 - 0.111{\text{V}}_{\text{D}} + 0.06$$

**Fig. 8:**
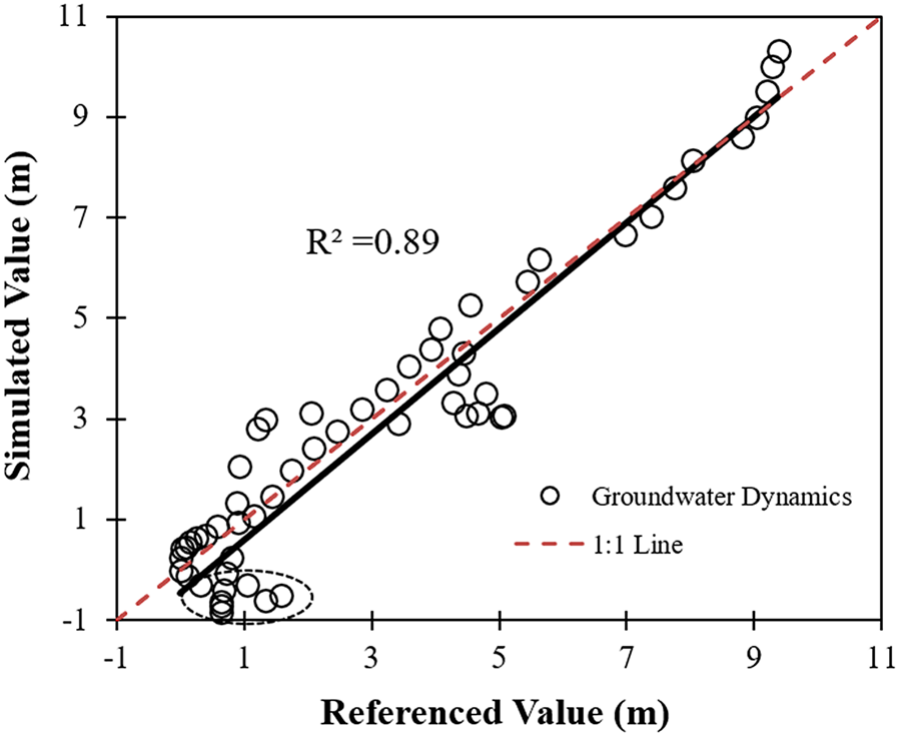
Calibration of the groundwater dynamics driven by oasis AET, scale and mountainous discharge

The regression revealed that the main factors influencing groundwater variation were oasis AET, oasis scale and water diversion by different kinds of water conservancy projects. The regression coefficient was calibrated to an R^2^ of 0.89 (Fig. 8), indicating that the relationship between the four variables (dependent variable was the monitored groundwater dynamics) was essential. A negative value indicates an increase in the groundwater level, generally corresponding to slight dynamics (points in the ellipse). Equation (7) is considered to express the relationship among the four variables with satisfactory reliability at a regional level.

#### Ideal oasis scale for a sustainable groundwater system

**Fig. 9:**
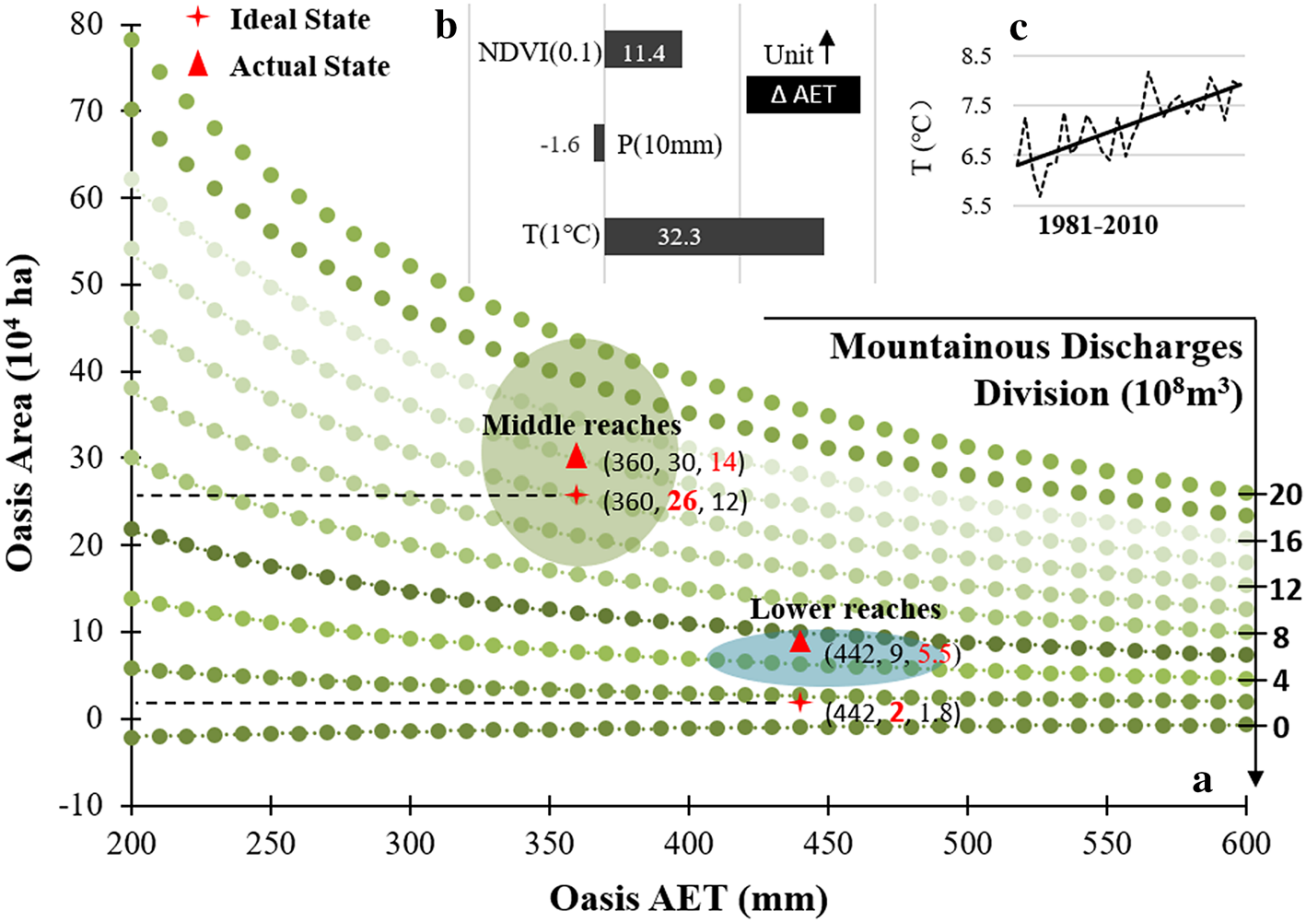
Ideal oasis water utilization based on consideration of oasis area, AET and mountainous discharges (**a**). Regional AET variations due to unit changes in influential factors (**b**) and increasing air temperature (**c**) in the SYRB during the 1981 to 2010 period are also presented

Under the framework illustrated in Eq. (7), the combination of irrigation area and oasis AET impacted the groundwater level drawdown, while the diversion of land surface water could counteract and retard the decline in the groundwater system. Given the neglect of local interactions between the land surface and underground water system (e.g., temporary exchanges of water in some of the river sections, local depressions due to intensive abstractions, evaporation in water bodies) and the induced errors in quantifying the relationships, the scenario of no groundwater drawdown could aid in further exploring the rational or ideal oasis water utilization under the background of human regulation and climate change. Figure 9 was plotted in correspondence to different surface water divisions under the hypothesis of no groundwater level drawdown, which could also aid in understanding oasis water utilization when considering groundwater system recovery. Ellipses present ranges of oasis AET and scale from 1981 to 2010 in the SYRB. Triangles indicate the average of the variables, while the crosses represent the ideally rational oasis area supported by the real land surface water diversion without groundwater drawdown. During the time period from 1981 to 2010, observations and simulations revealed numerical ranges of oasis AET (334–396, in mm) and oasis scale (18.4–45.0, in 10^4^ ha) in the middle reaches, while those in the lower reaches were in the ranges of 408–483, in mm and 4.5–11.1, in 10^4^ ha for oasis AET and scale, respectively (ellipse in Fig. 9a). Oasis AET and scale were averaged into to mm and 30 × 10^4^ ha in the period in the middle reaches, respectively, pointing to a virtual water diversion of 14 × 10^8^ m^3^ if there were no groundwater drawdown. This scenario was impossible because of policy-regulated water division to the lower reaches. The deficit was matched by groundwater abstractions. The situation was the same in the lower reaches, where the 30-year average oasis AET and scale were 442 mm and 9 × 10^4^ ha, respectively, corresponding to a requirement of 5.5 × 10^8^ m^3^ for land surface water division. This situation occurred in the 1950–1960s when the oasis scale in the middle reaches was relatively small. Real water diversion for oasis utilization in the middle reaches and lower reaches averaged approximately 12 × 10^8^ m^3^ and 1.8 × 10^8^ m^3^, respectively, from 1981 to 2010. If the decline in the groundwater system did not continue, the ideal oasis scale should be approximately 26 × 10^4^ ha and 2 × 10^4^ ha in the middle and lower reaches (bold red numbers in Fig. 9a), respectively, meaning that the ideal shrinkage of the oasis scale for a healthy groundwater system in the SYRB may be 11 × 10^4^ ha. Notably, the above analysis was based on the exclusion of V_LI_ when applying the module for discussion. Considering this part of water use would lead to more water consumption and a higher impact on the health of the underground water system.

## Discussion

### A 30-year basin water balance in the SYRB

**Fig. 10:**
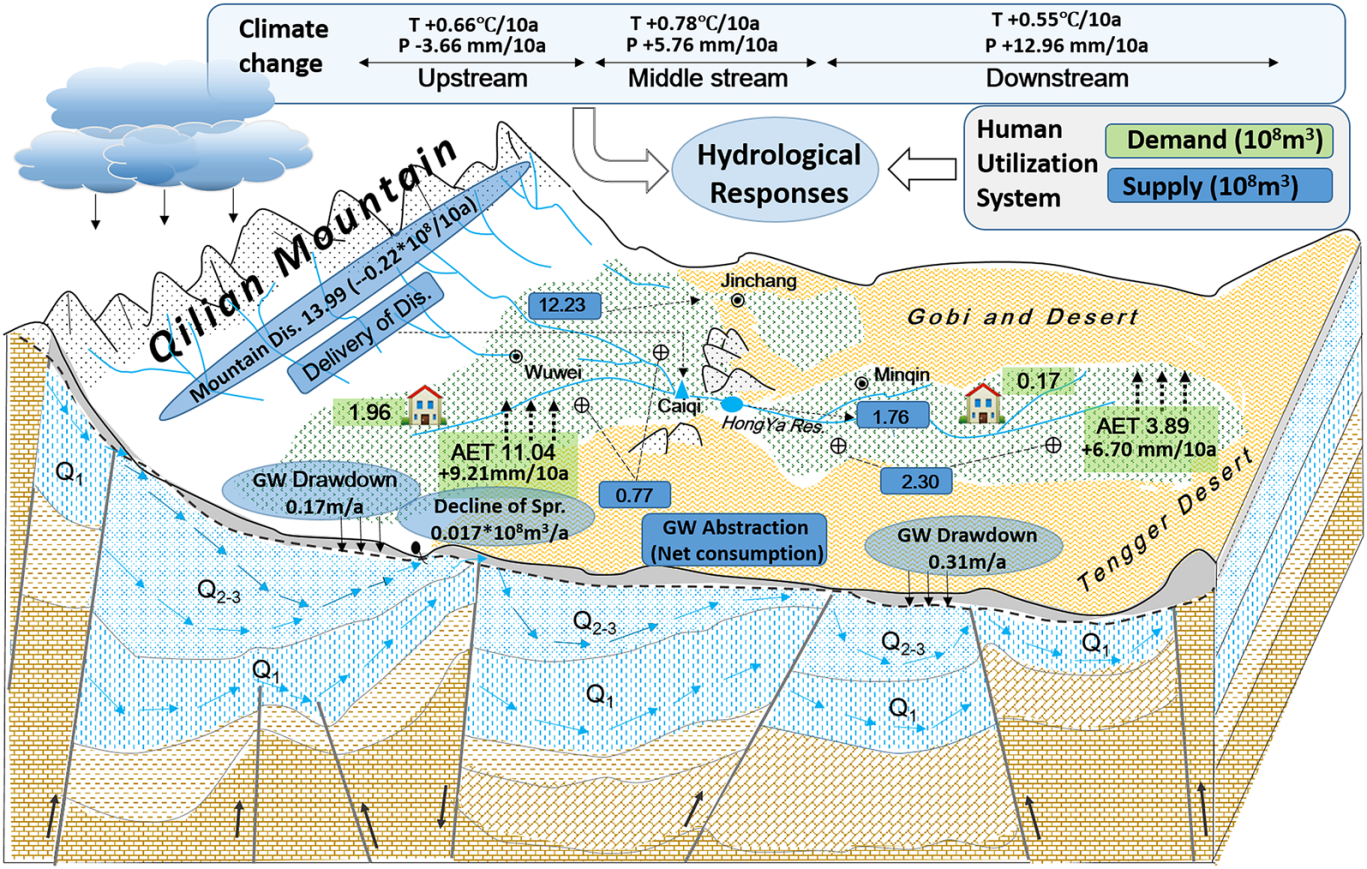
Conceptual illustration of groundwater dynamics corresponding to regional climate change and oasis water consumption across the SYRB during the 1981 to 2010 period

In the SYRB, mountainous discharges and abstractions of groundwater supported a large oasis AET requirement, combined with industrial water use and household water consumption. Sen’s slope was used to test T, which presented an overall increase across the whole basin (Fig. 9c), while P decreased in mountains and increased in plain areas during the 1981 to 2010 period. Under the background of climate change, mountainous discharges presented an overall decrease, while the extension of arable land led to an aggregation of land surface AET in the irrigated oasis (Fig. 10). Reservoir-canal-well systems, initially developed to support the large irrigation demand, led to dried-up river channels in addition to regional groundwater drawdown. Statistics in the SYRB revealed that the annual rate of groundwater drawdown in the lower area was at a high rate of 0.31 m/a, resulting in near-bare spring outflow for a relatively long time. The average drawdown rate was 0.17 m/a in the middle reaches. Spring outflow there declined at an annual rate of 0.017 × 10^8^ m^3^/a during that time period [60].

Mountain discharges from the upper river basins vary over time due to the effects of climate change on land surface water flux, while hydrological responses in the middle and lower plains are predominantly impacted by human activities. A decrease in mountainous discharges, along with an increase in the oasis AET requirement in the middle and lower reaches, not only led to depressions of spring outflow and phreatic evaporation but also to a reduction in necessary water absorption by roots [61]. Degeneration of land cover would lead to a higher risk of oasis destruction due to interior and circumjacent desertification. In particular, the high risk of oasis survival in the lower reaches of the SYRB has been verified over the past several decades, coinciding with desert extension in other areas and with strengthening dust storms in northwestern China [62]. These impacts indicate the negative results of overexploitation on water resources and its gradual consequences for regional hydrology and ecology; additionally, these impacts serve as a warning for pursuing economic well-being at the expense of the environment. Our study could be considered a 30-year case example when the above contradictions were remarkable in inland river systems in arid regions.

### Oasis and water management

For the large population (especially farmers) survival, resource shortage-induced water problems are still crucial to oasis health in the SYRB. Furthermore, an increase in air temperature, together with strengthened vegetation dynamics, facilitated regional AET requirements. According to this study, increasing precipitation suppressed oasis AET, and combined with a reduction in water utilization to some extent, the effectiveness was slight when compared with the former two factors. Under a background of warming and facing deterioration of the underground system, rational development of oasis soil and water needs more attention and better planning. In the late 2000s, strict rules were implemented to partition water resources between the middle and lower reaches (division part should not be less than 2.7 × 10^8^ m^3^ in a year [63], corresponding to a suitable oasis area of 4 × 10^4^ ha according to our study), to cut down groundwater abstraction to maintain a healthy underground system and to demonstrate advanced water-saving methods in experimental regions (which may lead to a decrease in the AET requirement). All of these methods have helped but not enough. On the one hand, we should further improve the crop types in oases by planting more low water consumption crops and reducing high water consumption crops. On the other hand, we can reduce cultivated land area, return farmland to forest and grassland, supervise ground water extraction and even close wells to protect the groundwater system. In addition, water diversion from outside the SYRB would be the key to achieving better socioeconomic development under the background of a changing climate.

## Conclusion

In this study, various-source data were used to calibrate and determine the driving factors that influence net oasis water consumption and regional groundwater dynamics in the selected SYRB of arid northwestern China. Module analyses revealed a warming-dominated AET increase, while oasis extension led to large amounts of water consumption and remarkable drawdown of regional groundwater during the 1981 to 2010 period. The drawdown rates of regional groundwater averaged 0.17 m/a and 0.31 m/a in the middle and lower reaches, respectively, indicating an approximately continuous decline in the basin groundwater system. From the perspective of groundwater system recovery, our study quantitatively pointed to prominent gaps between real oasis scales and rational scales in both the middle and lower reaches. For a sustainable future of population survival and land surface ecology in the SYRB, moderate shrinkage of the oasis scale and water diversion from outside into the basin would be essential.

## Data Availability

The gauge-based hydrometeorological observations are available in China’s National Meteorological Information Center database at http://www.cma.gov.cn/; hydrological observational data are supplied by the Gansu Province Bureau of Hydrology and Water Resources Monitoring; well-monitored groundwater data are from administrative units, including the Gansu Province Bureau of Hydrology and Water Resources Monitoring, Gansu Province Geology Survey, and Gansu Province Environmental Protection Agency; the NDVI that supports the findings of this study are available from the Advanced Very High Resolution Radiometer (https://ecocast.arc.nasa.gov/) and the Moderate Resolution Imaging Spectroradiometer (http://glovis.usgs.gov/); NLCD data are supported by China’s National Land Cover Dataset (http://www.resdc.cn/); and the GLEAM-AET data that support the findings of this study are available on the VU University Amsterdam Geoservices website (http://geoservices.falw.vu.nl).

## Abbreviations

SYRB: Shiyang River Basin
T: Temperature
P: Precipitation
NDVI: Normalized difference vegetation index
AET: Actual evapotranspiration
GLEAM: Global land evaporation Amsterdam model
AVHRR: Advanced very high resolution radiometer
MODIS: Moderate resolution imaging spectroradiometer
IDW: Inverse distance weighted
NLCD: China’s National Land Cover Dataset
DTM: Digital terrain mode
DEM: Digital elevation model
AOI: Area of interest
MVC: Maximum-value composite
Dis.: Discharge
GW: Groundwater
Spr.: Spring outflow
